# Practical Aspects of Medical Science

**Published:** 1894-03-17

**Authors:** 


### Practical Aspects of Medical Science.

Some very weighty arguments in favour of the beneficial results of a vegetable diet appeared recently in the pages of a contemporary. This subject is one we usually find treated from a narrow and sectarian point of view, from the standpoint of a vegetarian, in fact, but in the case of Mr. Mark Knapp, the author of the article to which we now refer, this is not so. Here we find the matter treated dispassionately and on common grounds, and the results of the writer's investigations are both suggestive and interesting. It should be understood at once that our authority in thus pointing out what are the exact physiological and pathological benefits which accrue to the human race from the use of vegetables as a diet by no means excludes meat from the ordinary *régime*. Mr. Knapp points out, "We can neither confine ourselves to the exclusive use of meats, nor to that of vegetables, they are of like importance to sustain our equilibrium of health or life . . . The chemical constituents that meats have, vegetables have not; and even if they have like quality, they do not have like quantity. The proportions differ in different kinds of meat and in different kinds of vegetables; and if we have enough of nitrogenous substances in a pound of meat to supply waste of one structure, we lack the proper amount of earthy salts to supply other tissues." In England, certainly, we do not find that sufficient attention is paid to the necessities of supplying these elements or compounds which are needed by our system. Motives, more due to thrift and economy than to physiology, have altered this state of things with other nations. Surely it is our duty to give more consideration to the importance of strengthening and fortifying our framework. König, the great German authority on this subject, urges the vital necessity for so doing. "For the growing organism earthy phosphates," he writes, "are of great importance, for out of these the bony skeleton is made up."

If it is not nutrition, what is it then which influences the height of these animals? The differences, too, of appearance and character amongst those "children of Nature" upon whom civilisation and progress of science cannot pour its enlightening rays? our authority asks, dismissing from his mind all other factors than food to explain stature.

He gives us, again, further illustration in defence of his theory in the instance of the build of two primitive races, the one from the North, the Samoyede, the other from the South, the Polynesian, two tribes as diametrically opposed in diet as they are in locality. In the case of the Polynesians, a tall, well built-up race, their diet is predominently of a vegetable nature; roots of ferns, berries, cabbage shoots, bread-fruit, and sweet potatoes forming the principal part. The Samoyede, a tribe of Northern Siberia, are, on the other hand, remarkable for their shortness of stature. These people having little assistance from agricultural products to assist their supplies, depend mainly for their food on the results of their fishing and hunting. "Not only," concludes our authority, "does the animal kingdom give us more than sufficient proof of the fallacy of any other theory than diet as the cause of height, also the ethnology of man, who has not yet been brought into intimate connection with the civilised world, proves it with certainty and positiveness."

Mr. Knapp's arguments in favour of this theory, which appears to be definitely established in his own mind, have a certain scientific foundation to start with. It is pretty certain that we do not pay sufficient attention in our dietry to the value of vegetables. If there are slight discrepancies in the examples he chooses to illustrate his subject it is barely to be wondered at. What has our authority to say to the respective stature and food of ancient Britons for instance? Surely they were not a nation of giants; they were short individuals in those early times, and yet they lived upon the *régime* set forth in this prescription which ensures height. And, again, in the case of the Patagonians, are they purely a vegetarian race? This monster tribe are little if they are not hunters, subsisting on the produce of the chase. Mr. Knapp has given his subject serious study, but we think that there are other circumstances to be taken into account besides the circumscribed aid of food, which entails a physiological cost.

